# Association between systemic immune-inflammation index and 10-year risk of cardiovascular disease in the United States (NHANES 1999–2018)

**DOI:** 10.3389/ebm.2025.10704

**Published:** 2025-08-21

**Authors:** Yapan Yang, Runqi Tu, Lijie Zhu, Guian Xu, Tingjie Yang, Qingman Li, Che Wang, Honghui Yang

**Affiliations:** ^1^ Department of Cardiology, Central China Fuwai Hospital, Central China Fuwai Hospital of Zhengzhou University, Henan Provincial People’s Hospital Heart Center, Zhengzhou, Henan, China; ^2^ Department of Cardiology, Henan Provincial People’s Hospital, People’s Hospital of Zhengzhou University, Zhengzhou, Henan, China; ^3^ Department of Epidemiology and Biostatistics, College of Public Health, Zhengzhou University, Zhengzhou, Henan, China

**Keywords:** systemic immune inflammation index, cardiovascular disease, framingham cardiovascular risk scores, 10-year cardiovascular disease risk, inflammation

## Abstract

The relationship between the systemic immune-inflammation index (SII) and the risk of developing cardiovascular disease (CVD) over the next 10 years in the United States is largely unknown. The aim of this study is to assess the association between SII and 10-year CVD risk. This population-based cross-sectional study included 9901 participants aged between 30 and 74 from the National Health and Nutrition Examination Survey (NHANES) 1999–2018. The 10-year CVD risk was calculated using the Framingham cardiovascular risk score (FRS). The Pearson test, generalized linear model (GLM) and restricted cubic splines (RCS) were used to analyze the associations between SII and the FRS. Based on the total population, the Pearson test and GLM revealed that there were positive relationships between Ln-transformed SII (Ln (SII)) and the FRS. After adjusting for confounding factors, the odds ratio (OR) for the FRS was 1.52 (95% confidence interval [CI]: 1.12–2.06) per unit increment in Ln (SII) (*P* = 0.009). Compared to the lowest quartile (Q1) of Ln (SII), the OR for the FRS in the highest quartile (Q4) was 1.89 (95% CI: 1.20–2.98; *P* = 0.007). RCS revealed that there was a linear association between Ln (SII) and the FRS (*P* for non-linearity = 0.972). As Ln (SII) increased, the value of FRS rose gradually (*P* for overall trend <0.001). However, the relationship between Ln (SII) and FRS showed ethnic heterogeneity. In conclusion, SII exhibits significant associations with 10-year CVD risk as assessed by the FRS. However, this association varies across ethnic groups, necessitating cautious application and further validation.

## Impact statement

This study investigated the relationship between systemic immune-inflammation index (SII) and cardiovascular disease (CVD) risk. The 10-year CVD risk was calculated by Framingham cardiovascular risk scores (FRS). We found that there was a positive significant association between SII and 10-year CVD risk. Therefore, SII is expected to become an effective metric for identifying the 10-year CVD risk of human, providing well preventive strategies and improving risk stratification.

## Introduction

Cardiovascular disease (CVD) is one of the most significant public health problems threatening human life. In recent years, it has remained the leading cause of death and disability worldwide in addition to being the leading cause of disease burden in the United States [1]. A report from the American Heart Association suggested that the prevalence of CVD is 48.6% in adults aged ≥20 years. Moreover, in 2020 the number of cardiovascular deaths worldwide was 19.05 million, which amounted to an increase of nearly one-fifth since 2010 [2]. Dyslipidemia, hypercoagulability, insulin resistance, hypertension, and inflammatory responses are the risk factors for the pathogenesis of CVD [3]. Based on several sex-specific multivariable risk factors, the Framingham Heart Study developed the first CVD risk equations, which were used to quantify risk and guide preventive care [4]. The Framingham Risk Score (FRS) is a widely used predictive tool that can be applied to relatively healthy individuals to estimate their probability of having a fatal or non-fatal cardiovascular event over the next decade [5, 6], and it has been used in several studies [6–8]. In addition, in recent years, inflammatory cytokines have shown promise as diagnostic tools for coronary heart disease, heart failure (HF), and other CVDs [9, 10].

With the in-depth study of chronic systemic inflammation, it has been found that inflammatory responses are connected to many different diseases [11–14]. Several immune cell types and inflammatory mediators have been implicated in the progression and pathogenesis of CVD [15, 16]. Mounting evidence suggests that chronic inflammation substantially contributes to cardiovascular risk. The Systemic Immune-Inflammation Index (SII), which is calculated using platelet, neutrophil, and lymphocyte counts, is a comprehensive biomarker that reflects both local immune responses and systemic inflammation [17]. Mechanistically, SII captures key inflammatory processes across atherogenesis. In the early phase, hemodynamic stress and lipid abnormalities appear to trigger inflammatory activation in endothelial cells, facilitating monocyte recruitment through adhesion molecules [18]. In the advanced phase, macrophage-derived inflammatory mediators promote extracellular matrix degradation via matrix metalloproteinases, increasing plaque vulnerability [19, 20]. Although SII has been established as an independent predictor of cancer, CVD and all-cause mortality [21–26], its association with the FRS — a key CVD risk assessment tool — remains unclear.

Previous studies on SII and CVD risk have several limitations. First, existing analyses have primarily assumed linear relationships, potentially overlooking complex nonlinear associations between SII and CVD outcomes. Second, there has been insufficient consideration of key lifestyle confounders, particularly comprehensive adjustment for physical activity patterns and dietary factors. Third, the integration of SII with established clinical risk prediction tools like the FRS remains unexplored in population-based studies. To address these limitations, our study specifically: (1) employs restricted cubic splines (RCS) to characterize potential non-linear relationships, (2) incorporates enhanced adjustment for objectively measured lifestyle factors including device-based physical activity and dietary intake, and (3) evaluates the relationship between SII and the FRS in a nationally representative sample. This study was based on the National Health and Nutrition Examination Survey (NHANES) to assess the association of SII with the FRS.

## Materials and methods

### Data and sample source

The NHANES is a cross-sectional survey conducted by the National Center for Health Statistics (NCHS) in the United States. The NCHS and the Centers for Disease Control and Prevention (CDC) conducted the survey. The NCHS’ Research Ethics Review Board evaluated and approved the NHANES study protocol. The study protocol was approved by the NCHS Ethics Review Board, and all participants provided informed consent. For further confirmation, please refer to the link to the NCHS ethics approval document for the NHANES data^1^. Briefly, the NHANES uses a stratified and multistage probability approach, which surveys approximately 5,000 participants annually. The data collected includes demographic data, questionnaire data, laboratory data, examination data and limited access data.

**FIGURE 1:**
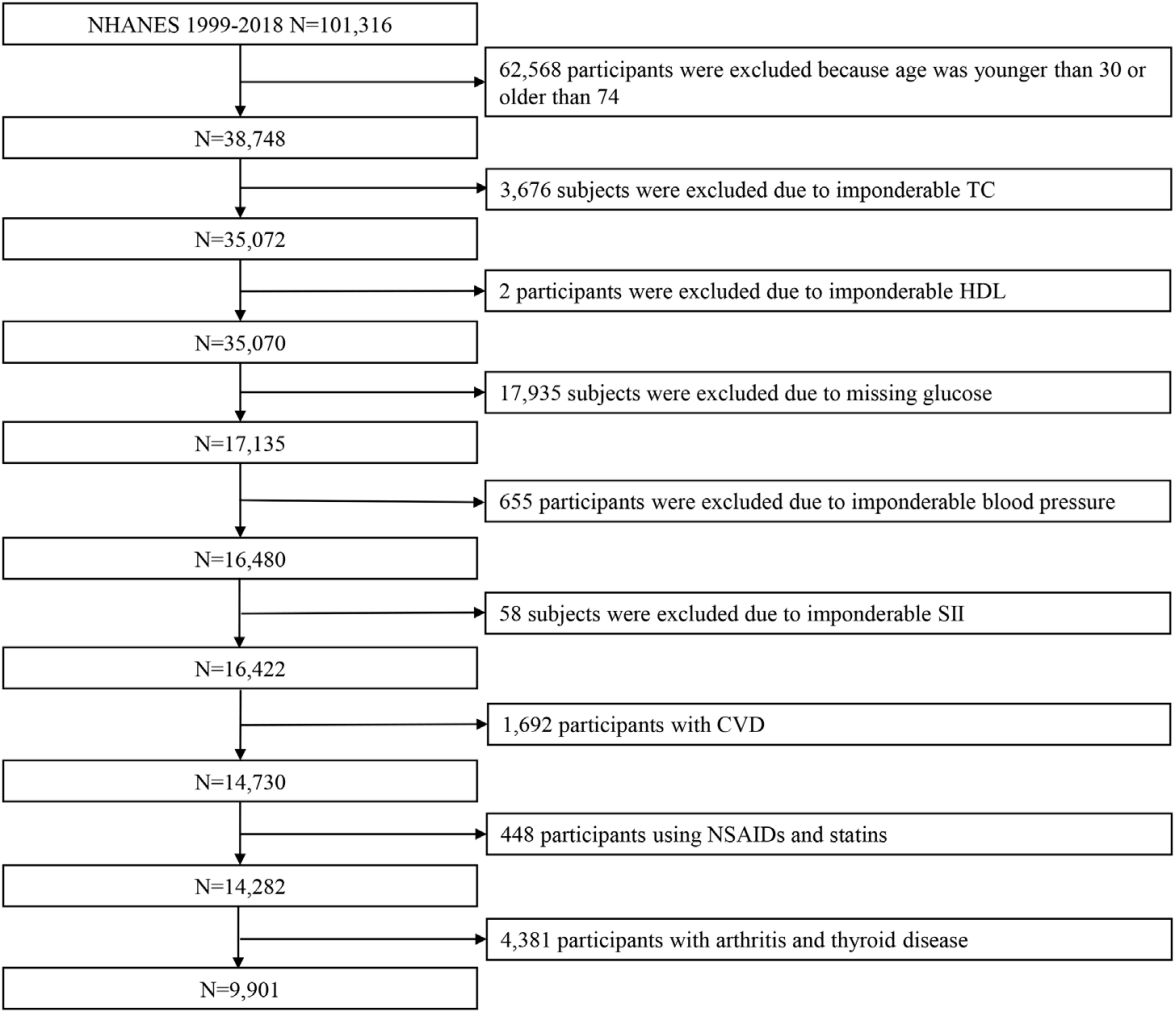
Flowchart showing the selection of participants from the NHANES from 1999 to 2018. Abbreviations: NHANES, National Health and Nutrition Examination Survey; TC, total cholesterol; HDL, high-density lipoprotein; SII, systemic immune-inflammation index; CVD, cardiovascular disease.

There were a total of 101,316 participants included in the NHANES from 1999 to 2018. Of those, we excluded 62,568 participants because their age was younger than 30 or older than 74; 3,676 subjects were excluded due to imponderable total cholesterol (TC); 2 individuals were excluded due to imponderable high-density lipoprotein (HDL); 17,935 were excluded due to missing glucose; 655 participants were excluded due to imponderable blood pressure; and 58 individuals were excluded due to imponderable SII. Of 1,692 participants with CVD,448 were excluded for using NSAIDs and statins; 4,381 subjects with arthritis and thyroid disease were excluded. Finally, 9901 participants were included in this study (Figure 1).

### Systemic immune-inflammation

The SII is based on a complete blood count. Standardized protocols for measuring blood counts (platelets, lymphocytes, and neutrophils) were provided by the NHANES Laboratory Procedures Manual. More details can be found at^2^. In the present study, the following equation was utilized to calculate the SII (SII = platelet count × neutrophil count ⁄ lymphocyte count) [17]. The SII data were unevenly distributed and skewed to the right. Natural logarithmic transformation converts absolute differences into proportional changes. For clinical interpretation, Ln-transformed SII (Ln(SII)) values can be back-transformed to the original scale. A one-unit increase in Ln (SII) corresponds to an e-fold (≈2.72-fold) multiplicative change in the original SII values. Based on this, the SII data need to be Ln-transformed. Supplementary Figure S1 shows the distributional characteristics and normality assessment of SII values. (A) The histogram of the raw SII values showed extreme right-skewness. (B) The quantile-quantile plot confirmed severe non-normality (Anderson-Darling A = 617.20, *P* < 0.001). (C) The histogram of Ln (SII) showed substantial improvement, although with residual skewness. (D) The quantile-quantile plot of the transformed values demonstrated a persistent deviation from normality (A = 6.62, *P* < 0.001). The red lines represent theoretical normal distributions.

### FRS

The FRS is a general 10-year risk estimate for CVD, which was developed using a Cox model based on the Framingham Heart Study [4]. In the present study, the FRS was calculated using sex, age, TC, HDL, systolic blood pressure (SBP), treatment for hypertension, smoking and diabetes. The FRS was Ln-transformed to reduce the effects of non-normality. To eliminate the potential contribution of neighborhood clustering by age and sex on neighborhood-level variance, the outcome variable used in the analysis was the normalized residual of the Ln-transformed FRS regressed on age and sex. Low 10-year CVD risk was defined as FRS <10%; FRS ≥10% was considered intermediate or high 10-year CVD risk.

### Covariates

The baseline characteristics included demographic factors (age, sex (men or women), education (high school or below and college graduate or above), marital status (married/Living with a partner, never married and widowed/divorced/separated), poverty income ratio (PIR) (2, 2-4 and 4) and ethnicity (Mexican American, Other Hispanic, Non-Hispanic White, Non-Hispanic Black, and Other Ethnicity), behavioral factors (smoking, alcohol consumption, physical activity (PA), and energy intake), and low-density lipoprotein (LDL). The metabolic equivalent (MET) represents the rate of oxygen uptake required to maintain the body’s basic metabolic processes while at complete rest. In line with World Health Organization (WHO) recommendations, we used MET values of 3.3 for walking, 4 for moderate activities, and 8 for vigorous activities [27]. PA was valued by total MET-minutes/week. Vigorous activity was defined as accumulating at least 3,000 MET-minutes/week, while moderate activity required a minimum of 600 MET-minutes/week. Individuals who met neither criterion were classified into the light activity category. Energy intake was estimated using the dietary intake data from the 24-h period before the interview. Body mass index (BMI) was calculated by dividing weight in kilograms by the square of height in meters. Participants were categorized into four BMI groups according to WHO criteria: underweight (BMI <18.5 kg/m^2^), normal weight (BMI 18.5–24.9 kg/m^2^), overweight (BMI 25.0–29.9 kg/m^2^), and obese (BMI ≥30.0 kg/m^2^). The diagnosis of CVD was determined by self-reported physician diagnoses obtained during interviews using a standardized questionnaire. subjects were asked, “Has a doctor or other health expert ever informed you that you have angina/congestive heart failure/coronary heart disease/heart attack (myocardial infarction)/stroke?” If the answer was “yes” to any of the above questions, the subjects were considered to have CVD.

### Statistical analysis

Participants were stratified into four groups according to Ln (SII) quartiles. Continuous and categorical baseline characteristics were presented as mean ± standard deviation (SD) and number (percentage), respectively. Differences in the distribution of variables were compared using a weighted one-way analysis of variance (ANOVA) test for continuous data and a weighted chi-square test for categorical data, respectively.

The Pearson test was used to analyze the correlation between Ln (SII) and the FRS. Two models were developed to assess associations of Ln (SII) and the FRS or 10-year CVD risk level by using weighted GLM: the crude model was not adjusted; the adjusted model was adjusted for education, marital status, PIR, ethnicity, alcohol consumption, PA, energy intake, BMI, and LDL. To rigorously assess the robustness of our results, we performed a subgroup analysis to investigate potential modifying effects based on ethnicity.

Furthermore, weighted RCS were used to explore non-linear relationships between Ln (SII) and FRS or 10-year CVD risk level. Knot placement and model specification were implemented using Harrell’s rms package. Interior knots were positioned at clinically relevant percentiles of the Ln (SII) distribution following established conventions: the 10th, 50th, and 90th percentiles were used for the optimal 3-knot configuration, which was determined by comparing Akaike Information Criterion (AIC) across 3 to 6 knot models. Boundary knots were automatically anchored at the observed minimum Ln (SII)value of 2.47.

All statistical analyses were performed using R version 4.2.2, with a significance threshold of *P* < 0.05, and all statistical tests were two-sided.

## Results

### Study population characteristics

A total of 9901 subjects were included in the study, among whom 53.27% were men, and 46.73% were women. The number of participants who were judged as having an intermediate or high 10-year CVD risk was 1,438 (14.52%). The demographic characteristics of the subjects by Ln (SII) quartiles are shown in Table 1. The results suggest statistically significant differences in age, gender, marital status, ethnicity, smoking status, treatment for hypertension, BMI, PA, TC, FRS, and 10-year CVD risk level (all *P* < 0.05). The demographic characteristics of the subjects after weighting are shown in Supplementary Table S1.

**TABLE 1:** Demographic characteristics of subjects (n = 9901) in the NHANES 1999–2018.

Variables	Q1 (N = 2,476)	Q2 (N = 2,475)	Q3 (N = 2,475)	Q4 (N = 2,475)	Total (N = 9901)	*P*
Age (year) (mean ± SD)	48.02 ± 12.10	47.55 ± 12.22	46.99 ± 11.69	46.82 ± 12.15	47.34 ± 12.05	0.002
Gender (%)						<0.001
Men	1,500 (60.58)	1,404 (56.73)	1,275 (51.52)	1,095 (44.24)	5,274 (53.27)	
Women	976 (39.42)	1,071 (43.27)	1,200 (48.48)	1,380 (55.76)	4,627 (46.73)	
Education (%)						0.940
High school or below	1,190 (48.06)	1,181 (47.72)	1,170 (47.27)	1,183 (47.80)	4,724 (47.71)	
College graduate or above	1,285 (51.90)	1,291 (52.16)	1,303 (52.65)	1,291 (52.16)	5,170 (52.22)	
Marital status (%)						0.002
Married/Living with partner	1710 (69.06)	1726 (69.74)	1714 (69.25)	1,605 (64.85)	6,755 (68.23)	
Never married	423 (17.08)	444 (17.94)	431 (17.41)	526 (21.25)	1824 (18.42)	
Widowed/Divorced/Separated	322 (13.00)	275 (11.11)	303 (12.24)	315 (12.73)	1,215 (12.27)	
PIR (%)						0.569
<2	982 (39.66)	943 (38.10)	941 (38.02)	958 (38.71)	3,824 (38.62)	
2–4	635 (25.65)	602 (24.32)	623 (25.17)	636 (25.70)	2,496 (25.21)	
≥4	646 (26.09)	722 (29.17)	706 (28.53)	673 (27.19)	2,747 (27.74)	
Ethnicity (%)						<0.001
Mexican American	459 (18.54)	542 (21.9)	528 (21.33)	489 (19.76)	2018 (20.38)	
Other Hispanic	213 (8.60)	242 (9.78)	243 (9.82)	230 (9.29)	928 (9.37)	
Non-Hispanic White	690 (27.87)	952 (38.46)	1,062 (42.91)	1,178 (47.60)	3,882 (39.21)	
Non-Hispanic Black	778 (31.42)	465 (18.79)	403 (16.28)	373 (15.07)	2019 (20.39)	
Other Ethnicity	336 (13.57)	274 (11.07)	239 (9.66)	205 (8.28)	1,054 (10.65)	
Smoke (%)						<0.001
No	1,459 (58.93)	1,427 (57.66)	1,397 (56.44)	1,256 (50.75)	5,539 (55.94)	
Yes	1,017 (41.07)	1,048 (42.34)	1,078 (43.56)	1,219 (49.25)	4,362 (44.06)	
Alcohol consumption (%)						0.567
No	1709 (69.02)	1730 (69.90)	1734 (70.06)	1710 (69.09)	6,883 (69.52)	
Yes	568 (22.94)	566 (22.87)	574 (23.19)	595 (24.04)	2,303 (23.26)	
Treatment for hypertension (%)						0.034
No	2060 (83.20)	2033 (82.14)	2082 (84.12)	2009 (81.17)	8,184 (82.66)	
Yes	416 (16.80)	442 (17.86)	393 (15.88)	466 (18.83)	1717 (17.34)	
T2DM (%)						0.356
No	1,232 (49.76)	1,244 (50.26)	1,250 (50.51)	1,291 (52.16)	5,017 (50.67)	
Yes	1,244 (50.24)	1,231 (49.74)	1,225 (49.49)	1,184 (47.84)	4,884 (49.33)	
BMI (kg/m2) (mean ± SD)	27.98 ± 5.67	28.28 ± 5.79	28.78 ± 6.19	29.07 ± 6.80	28.53 ± 6.14	<0.001
Underweight	34 (1.37)	36 (1.45)	28 (1.13)	39 (1.58)	137 (1.38)	<0.001
Normal weight	742 (29.97)	714 (28.85)	677 (27.35)	671 (27.11)	2,804 (28.32)	
Overweight	612 (24.72)	570 (23.03)	559 (22.59)	526 (21.25)	2,267 (22.90)	
Obese	1,068 (43.13)	1,147 (46.34)	1,195 (48.28)	1,209 (48.85)	4,619 (46.65)	
Energy intake (kcal) (mean ± SD)	2,154.30 ± 945.14	2,152.5 ± 860.88	2,137.35 ± 924.32	2,137.03 ± 898.14	2,145.27 ± 907.38	0.859
PA (MET-minutes/week) (mean ± SD)	4,286.55 ± 8,987.26	3,658.71 ± 6,222.36	3,848.23 ± 15,673.21	2,993.56 ± 8,034.72	3,700.21 ± 10,367.76	0.001
Vigorous	625 (25.24)	673 (27.19)	731 (29.54)	799 (32.28)	2,828 (28.56)	<0.001
Moderate	742 (29.97)	750 (30.30)	719 (29.05)	700 (28.28)	2,911 (29.40)	
Light	666 (26.90)	606 (24.48)	561 (22.67)	490 (19.80)	2,323 (23.46)	
TC (mg/dL) (mean ± SD)	198.76 ± 40.91	199.81 ± 40.89	200.95 ± 39.55	201.96 ± 41.91	200.37 ± 40.84	0.035
HDL (mg/dL) (mean ± SD)	53.79 ± 16.46	52.97 ± 16.01	53.18 ± 16.24	53.64 ± 16.24	53.40 ± 16.24	0.249
LDL (mg/dL) (mean ± SD)	119.43 ± 35.18	120.31 ± 35.86	120.32 ± 33.9	120.32 ± 35.24	120.09 ± 35.05	0.779
SBP (mm Hg) (mean ± SD)	123.09 ± 18	122.61 ± 17.71	122.53 ± 17.87	122.68 ± 18.23	122.73 ± 17.95	0.695
Ln (SII) (mean ± SD)	5.46 ± 0.32	5.96 ± 0.09	6.28 ± 0.10	6.78 ± 0.28	6.12 ± 0.53	<0.001
FRS (mean ± SD)	5.04 ± 8.12	5.21 ± 7.89	5.29 ± 8.26	5.78 ± 8.61	5.33 ± 8.23	0.011
Low 10-year CVD risk	2,136 (86.27)	2,118 (85.58)	2,136 (86.30)	2073 (83.76)	8,463 (85.48)	0.032
Intermediate and high 10-year CVD risk	340 (13.73)	357 (14.42)	339 (13.70)	402 (16.24)	1,438 (14.52)	

Abbreviations: SD, standard deviation; PIR, poverty income ratio; SBP, systolic blood pressure; T2DM, type 2 diabetes mellitus; BMI, body mass index; PA, physical activity; MET, metabolic equivalent; TC, total cholesterol; HDL, high-density lipoprotein; LDL, low-density lipoprotein; Ln (SII), Ln-transformed SII; SII, systemic immune-inflammation index; FRS, framingham cardiovascular risk score; CVD, cardiovascular disease.

### Associations of Ln (SII) with the FRS

**FIGURE 2:**
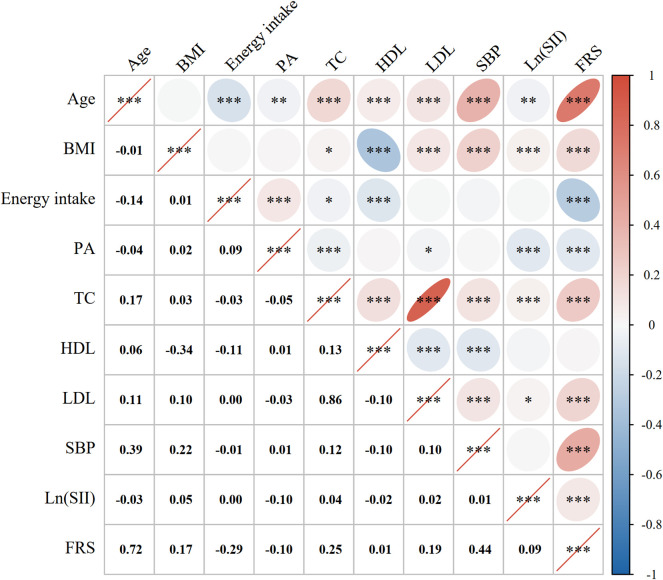
Correlation analysis. Pearson correlation coefficients between Ln (SII) with the FRS and the factors that were computed for the FRS. The correlation coefficients are shown as numbers and colors. Blue indicates a positive correlation and red indicates a negative correlation. A flatter circle represents a stronger correlation. *indicates *p* < 0.05; **indicates *p* < 0.01; ***indicates *p* < 0.001. Abbreviations: BMI, body mass index; PA, physical activity; TC, total cholesterol; HDL, high-density lipoprotein; LDL, low-density lipoprotein; SBP, systolic blood pressure; Ln (SII), Ln-transformed SII; SII, systemic immune-inflammation index; FRS, Framingham cardiovascular risk score.

A Pearson correlation analysis was performed to examine the correlation between Ln (SII) and the FRS, along with other continuous variables. There was a positive relationship found between Ln (SII) and the FRS, with a corresponding correlation coefficient of 0.09 (*P* < 0.001). Except for energy intake and PA, there were positive relationships between the other factors and the FRS. The results of the Pearson correlation coefficient are shown in Figure 2.

### Associations of Ln (SII) and Ln (SII) quartiles with the FRS

Table 2 suggests the results of the association between Ln (SII) and Ln (SII) quartiles and the FRS in the crude model and adjusted model. In the crude model, Ln (SII) was analyzed as a continuous variable. The odds ratio (OR) for FRS was 2.18 [95% confidence interval (CI): 1.57–3.02] per unit increment in Ln (SII). Compared to the lowest quartile (Q1) of Ln (SII), the OR (95% CI) for Q2, Q3, and Q4 were 1.22 (0.76-1.96), 1.86 (1.15–3.01), and 3.06 (1.90–4.93), respectively (*P* for trend <0.001). In the adjusted model, the OR (95% CI) for FRS was 1.52 (1.12–2.06) for a per-unit increment in Ln (SII). Compared to the Q1 of Ln (SII), the OR (95% CI) for Q2, Q3, and Q4 were 1.19 (0.73–1.92), 1.49 (0.95–2.35), and 1.89 (1.20–2.98), respectively (*P* for trend = 0.004).

**TABLE 2:** Associations of Ln (SII) and Ln (SII) quartiles with the FRS, as estimated by weighted generalized linear models.

Factor	Crude model			Adjusted model		
	OR	95%CI	*P* value	OR	95%CI	*P* value
Ln (SII)	2.18	1.57–3.02	<0.001	1.52	1.12–2.06	0.009
Stratified by Ln (SII) quartiles	1.47	1.26–1.71	<0.001	1.24	1.07–1.43	0.004
Q1	Ref			Ref		
Q2	1.22	0.76–1.96	0.417	1.19	0.73–1.92	0.488
Q3	1.86	1.15–3.01	0.013	1.49	0.95–2.35	0.086
Q4	3.06	1.90–4.93	<0.001	1.89	1.20–2.98	0.007

The crude model was not adjusted;

The adjusted model was adjusted for education, marital status, PIR, ethnicity, alcohol consumption, PA, energy intake, BMI, and LDL.

Abbreviations: Ln (SII), Ln-transformed SII; SII, systemic immune-inflammation index; FRS, framingham cardiovascular risk score; OR, odds ratio; Ref, reference; PIR, poverty income ratio; PA, physical activity; BMI, body mass index; LDL, low-density lipoprotein.

### The non-linear association between Ln (SII) and the FRS

**FIGURE 3:**
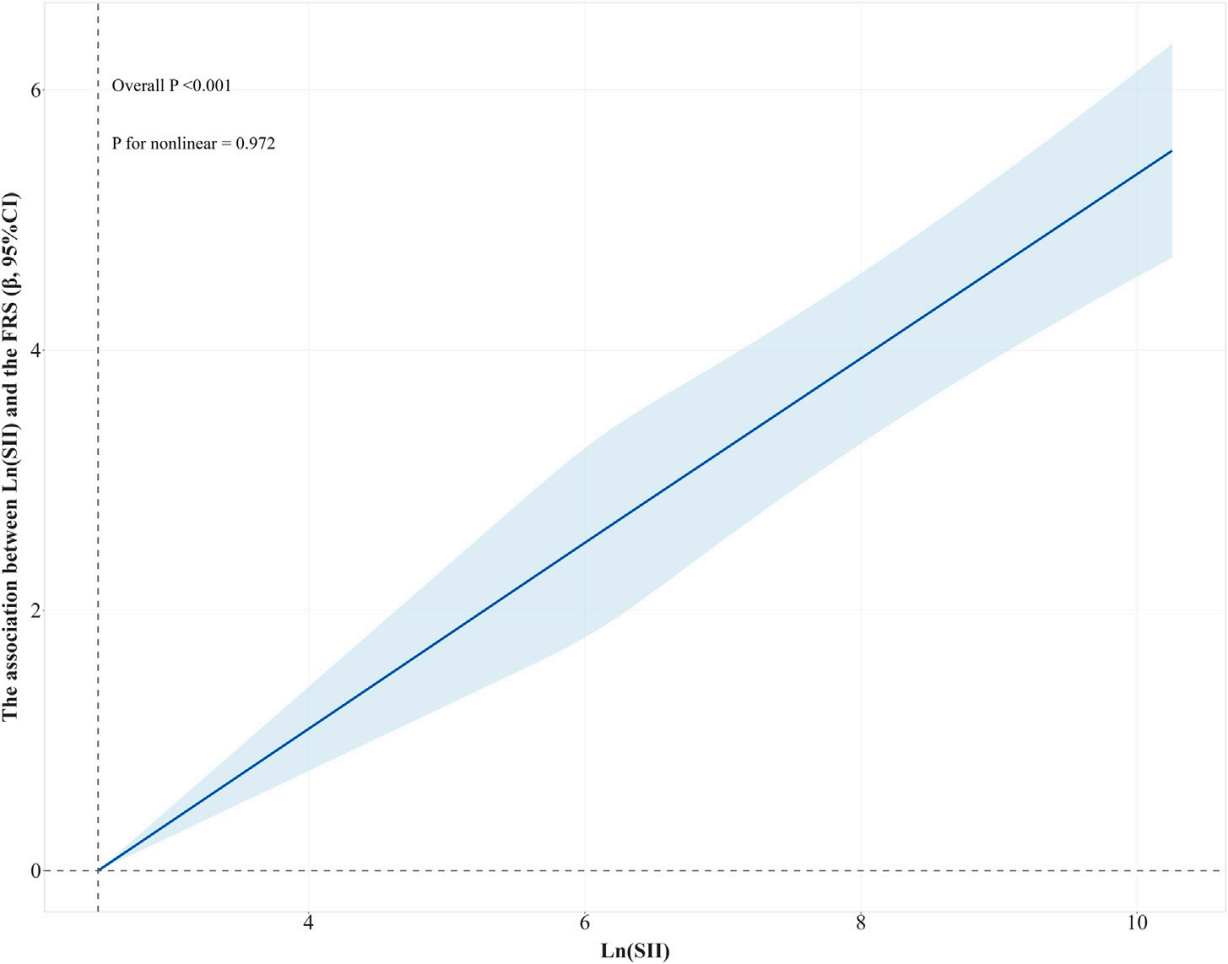
RCS curve of the association between Ln(SII) and the FRS. The results were adjusted for education, marital status, PIR, ethnicity, alcohol consumption, PA, energy intake, BMI, and LDL. Abbreviations: RCS, restricted cubic splines; Ln (SII), Ln-transformed SII; SII, systemic immune-inflammation index; FRS, Framingham cardiovascular risk score; PIR, poverty income ratio; PA, physical activity; BMI, body mass index; LDL, low-density lipoprotein.

A non-linear relationship was explored between Ln (SII) and the FRS by RCS. Figure 3 shows that a linear association was found between Ln (SII) and the FRS (*P* for non-linearity = 0.972). As Ln (SII) increased, so did the value of FRS (*P* for overall trend <0.001).

### The relationship between Ln (SII) and 10-year CVD risk

Supplementary Table S2 shows the results of the association between Ln (SII) and Ln (SII) quartiles and 10-year CVD risk in the crude model and adjusted model. After adjusting for the potential confounders, all associations became non-significant (OR range: 1.03–1.13, *P* > 0.05 for all comparisons). Supplementary Figure S2 shows a linear association between Ln (SII) and 10-year CVD risk (*P* for non-linearity = 0.541). With the Ln (SII) increasing, 10-year CVD risk gradually increased (*P* for overall trend <0.001).

### Subgroup analysis

Supplementary Tables S3-S12 present ethnicity-stratified analyses of the association between Ln (SII) (as both continuous and quartile variables) and the FRS and 10-year CVD risk level. After fully adjusting for education, marital status, PIR, alcohol consumption, physical activity, energy intake, BMI, and LDL, we observed distinct ethnicity-associated patterns: No significant associations were found among Mexican American patients (OR range: 0.84–1.75, all *P* > 0.05), Other Hispanic subjects (OR range: 0.34-1.39, all *P* > 0.05), or Non-Hispanic Black subjects (OR range: 0.98–2.34, all *P* > 0.05). In the Non-Hispanic White group, higher Ln (SII) was associated with an increased FRS (per-unit OR = 1.72, 95% CI: 1.13–2.60; Q4 vs. Q1 OR = 2.30, 95% CI: 1.27–4.16; *P* for trend = 0.007), but it was not associated with 10-year CVD risk (OR range: 1.05–1.18). Conversely, the Other Ethnicity group exhibited an inverse 10-year CVD risk association (per-unit OR = 0.46, 95% CI: 0.27–0.79; Q3 vs. Q1 OR = 0.41, 95% CI: 0.19–0.90), whereas no significant association was observed for the FRS (OR range: 0.48–0.86).

## Discussion

It is increasingly recognized that systemic inflammation initiates and exacerbates the pathological processes of chronic diseases. Many inflammatory predictors associated with CVD risk have been identified [28]. The present study showed that the FRS increased with the increase of SII. Both the Pearson correlation analysis and RCS revealed a significant positive association between Ln (SII) and the FRS. Therefore, SII may serve as a useful biomarker for assessing 10-year CVD risk in the general population. SII could be used to quickly identify high-risk subjects with a 10-year risk of CVD at a relatively low cost.

Several studies have supported the finding of a positive relationship between SII and the CVD risk [24, 25, 29]. Another study suggested that an elevation of SII level would be obvious in almost all subtypes of CVD, including ischemic stroke, hemorrhagic stroke, myocardial infarction, and peripheral arterial disease [30]. Furthermore, SII was found to be associated with poor short-term prognosis in atrial fibrillation patients with ischemic stroke [31]. Meanwhile, SII levels were also elevated in patients with ST-segment elevation myocardial infarction [32, 33]. Based on these, a close relationship was found to exist between SII and CVD risk. These results also provide sufficient evidence that chronic inflammation in the healthy population greatly increases the risk of developing CVD.

The underlying mechanisms of SII relating to the FRS can be attributed to several factors. First, chronic systemic inflammation can cause abnormal platelet aggregation, allowing them to adhere to the surface of endothelial cells, causing hypoxia, ischemia and microthrombus formation, leading to local tissue death [34, 35]. Second, long-term aberrant decrease of lymphocyte counts indicates excessive lymphocyte death in the human body, leading to reduced immune system capacity and immune dysfunction. Subsequently, lymphocyte death could further lead to endothelial dysfunction, abnormal aggregation of platelets, and thrombosis after platelet activation [24]. Third, monocytes and neutrophils can also promote abnormal coronary plaque status by activating and generating inflammatory responses, inducing atherosclerotic plaque rupture and thrombosis, thereby increasing the risk of adverse cardiovascular events [36]. As a complex inflammatory index, SII could effectively and comprehensively reflect the inflammatory state and immune system state of the body. The cumulative effect of the interaction between the three different cell lines synergistically enhances the association between systemic inflammation and the 10-year CVD risk assessed by the FRS. Therefore, the interaction between platelets, neutrophils, and lymphocytes may represent a potential therapeutic target for chronic inflammation in patients with high FRS.

Subgroup analyses revealed complex heterogeneity in the relationship between Ln (SII) and the FRS: significant positive associations were observed in Non-Hispanic Whites, whereas null effects were found in the Mexican American, Other Hispanic, and Non-Hispanic Black groups. In contrast, the Other Ethnicity group exhibited a negative relationship between Ln (SII) and 10-year CVD risk. These differential patterns likely reflect the interplay between ethnicity, SII and CVD. The findings in the Mexican American/Hispanic/Other Ethnicity groups may reflect the “health paradox” phenomenon [37, 38]. The reason for the different relationships between different ethnic groups likely originates from three interconnected mechanisms: First, racism generates population health disparities through the propagation of beliefs, attitudes, and treatment of group members by both individuals and institutions [39]. Second, some research suggests that Black individuals in the United States experience premature death from a variety of causes, including multiple diseases [40, 41]. Second, the immigrant health advantage has always played a critical role in this situation. Several studies have shown lower mortality rates [42], and fewer chronic conditions [43] in immigrants than in US-born subjects. Third, current ethnic classifications inadequately capture heterogeneity, while undifferentiated pan-ethnic labels mask critical variations in nativity and phenotype [44].

It is important to emphasize the limitations of the present study. First, the NHANES had a cross-sectional design, which limits its ability to establish causality. Specifically, simultaneous SII and FRS measurements prevent determining whether inflammation precedes or results from subclinical CVD; influencing factors (e.g., occult infections, undiagnosed autoimmune disorders) may independently influence both SII and CVD risk. Furthermore, single-time-point in front sampling cannot capture how the relationship between SII and CVD risk evolves over time. Considering the large sample size of the NHANES and its complex, multi-stage, probabilistic sampling design, the results still indicated stability in the relationship between SII and 10-year CVD risk. Second, due to missing information in the data set, excluded participants with incomplete data may introduce bias into the analysis. Third, hematological parameters were assessed at a single time point (a limitation inherent to the NHANES protocol), while the substantial sample size provides sufficient statistical power to detect meaningful population-level associations. Large-scale epidemiological investigations have consistently demonstrated that single measurements of inflammatory biomarkers retain significant predictive value for long-term cardiovascular risk stratification in adult populations. In addition, utilizing the NHANES database inevitably introduces the possibility of imprecise data capture and recall bias.

## Conclusion

The results demonstrate a significant positive association between SII and the FRS, supporting the potential of SII as an effective biomarker for identifying 10-year CVD risk. Nonetheless, variations in the relationship between SII and the FRS among different ethnic groups underscore the importance of careful application. Further studies with larger and more diverse cohorts are required for comprehensive validation.

## Data Availability

The datasets presented in this study can be found in online repositories. The names of the repository/repositories and accession number(s) can be found in the article/Supplementary Material.

## Footnotes

1
www.cdc.gov/nchs/nhanes/irba98.htm

2
https://www.cdc.gov/nchs/nhanes/biospecimens/serum_plasma_urine.htm
