# Supplementary figures and images for: Association between systemic immune-inflammation index and 10-year risk of cardiovascular disease in the United States (NHANES 1999–2018)
Source: Exp Biol Med (Maywood). 2025 Aug 21;250:10704. doi: 10.3389/ebm.2025.10704 (PMC12408401; doi:10.3389/ebm.2025.10704)

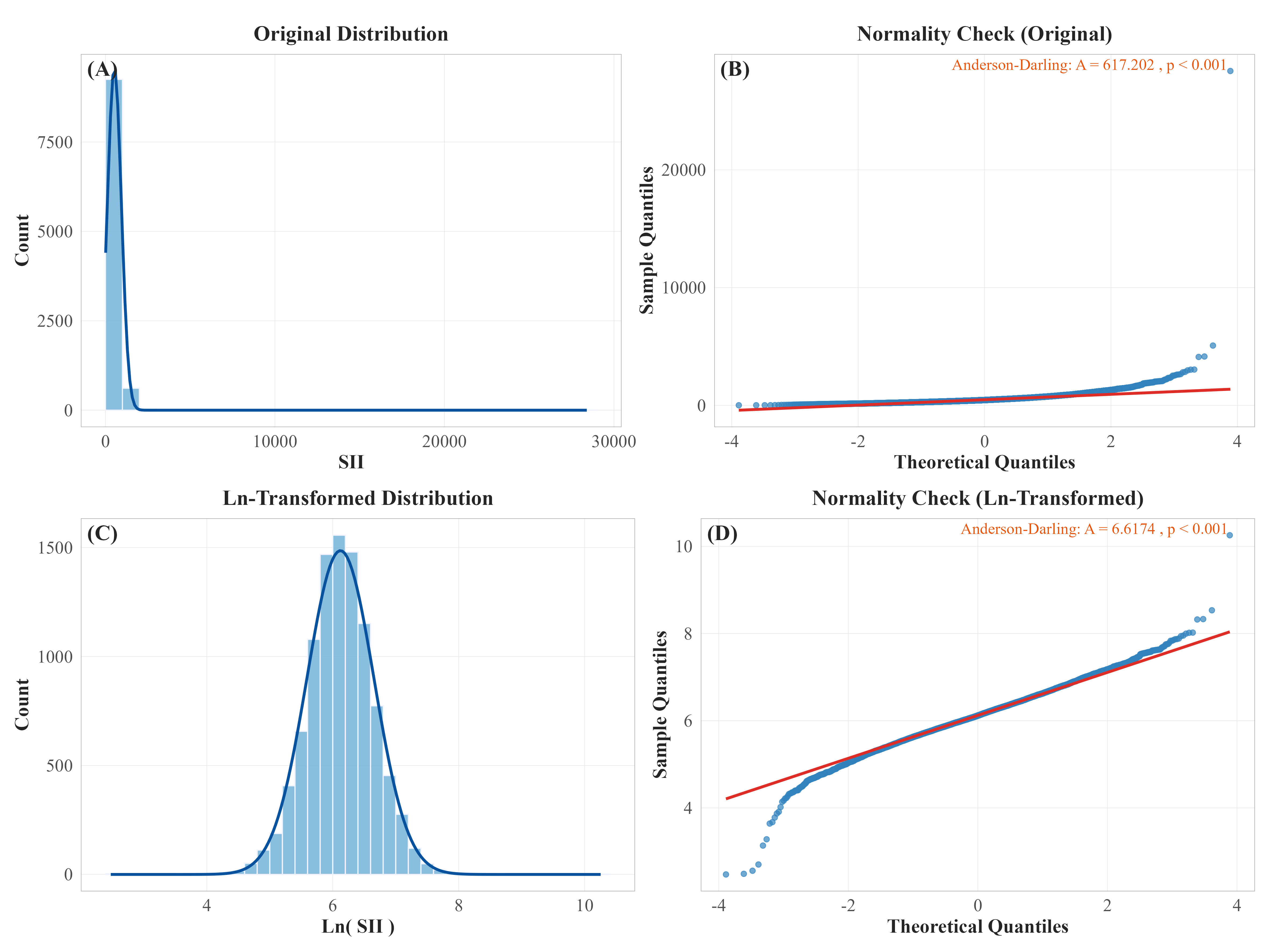

Supplement: Supplementary file 1 [file Image1.tiff]

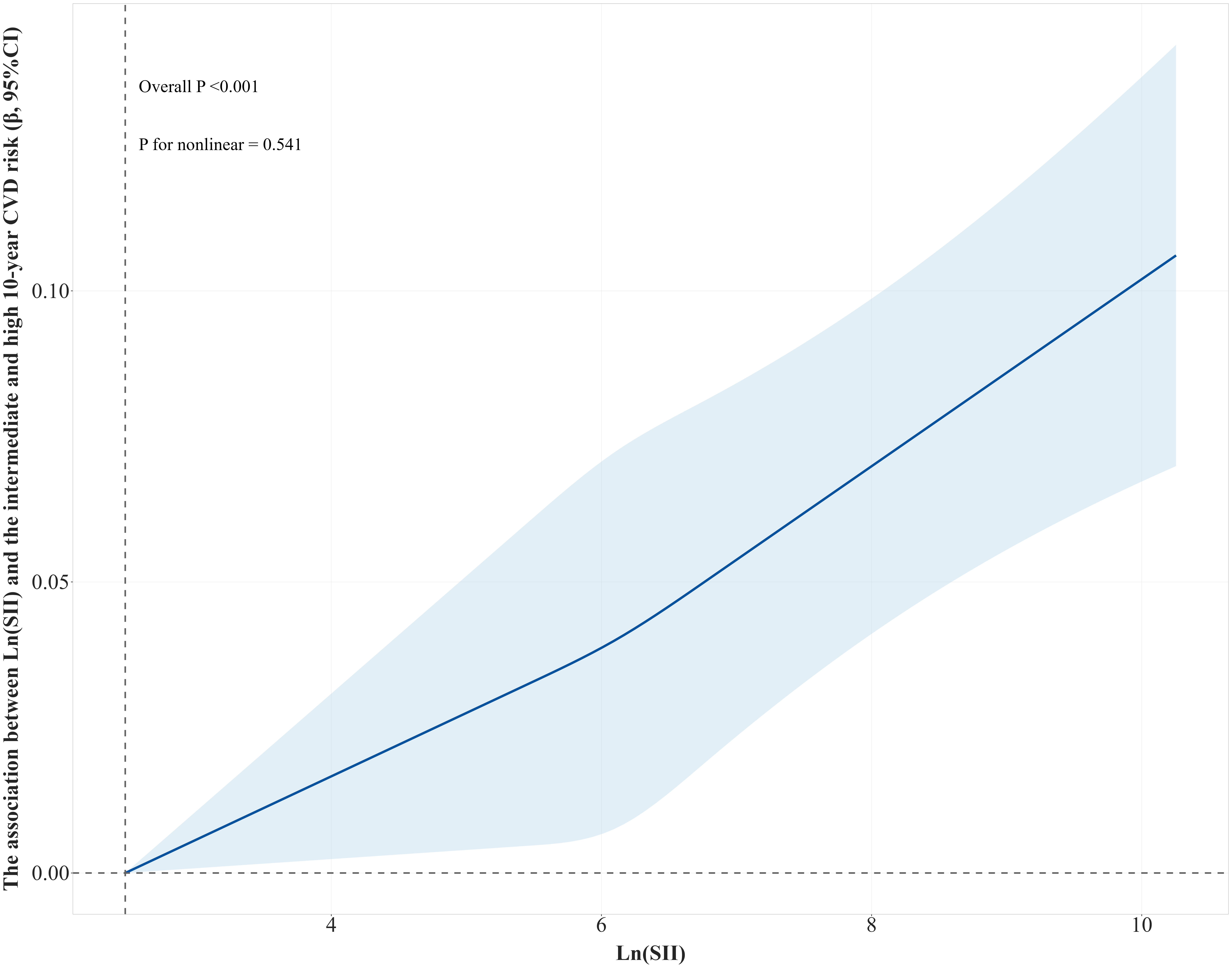

Supplement: Supplementary file 3 [file Image2.tiff]
