# Supplementary material for: Association between systemic immune-inflammation index and 10-year risk of cardiovascular disease in the United States (NHANES 1999–2018)
Source: Exp Biol Med (Maywood). 2025 Aug 21;250:10704. doi: 10.3389/ebm.2025.10704 (PMC12408401; doi:10.3389/ebm.2025.10704)
Supplement: Supplementary file 2 [file Supplementaryfile1.docx]

| **Supplemental Table1. Demographic characteristics of subjects after weighted in the NHANES 1999-2018** | | | | | | |
| --- | --- | --- | --- | --- | --- | --- |
| Variables | Q1 (N=207602430) | Q2 (N=236576992) | Q3 (N=245995864) | Q4 (N=251529353) | Total (N=941704638) | *P* |
| Age (year) (mean±SD) | 45.91±11.26 | 45.72±11.31 | 45.93±10.79 | 45.84±11.08 | 45.85±11.10 | 0.971 |
| Gender (%) |  |  |  |  |  | ＜0.001 |
| Men | 126029944(60.71) | 135622086(57.33) | 128668881(52.31) | 110130719(43.78) | 500451630(53.14) |  |
| Women | 81572486(39.29) | 100954906(42.67) | 117326982(47.69) | 141398635(56.22) | 441253008(46.86) |  |
| Education (%) |  |  |  |  |  | 0.953 |
| High school or below | 77069831(37.12) | 87448056(36.96) | 94943629(38.60) | 103192213(41.03) | 362653728(38.51) |  |
| College graduate or above | 130211802(62.72) | 148977654(62.97) | 150845712(61.32) | 148285935(58.95) | 578321103(61.41) |  |
| Marital status (%) |  |  |  |  |  | 0.002 |
| Married/Living with partner | 149794237(72.15) | 171714601(72.58) | 181382982(73.73) | 167839480(66.73) | 670731299(71.23) |  |
| Never married | 30119214(14.51) | 35651476(15.07) | 35612526(14.48) | 49516748(19.69) | 150899965(16.02) |  |
| Widowed/Divorced/Separated | 25220859(12.15) | 25322055(10.70) | 26069669(10.60) | 30133655(11.98) | 106746239(11.34) |  |
| PIR (%) |  |  |  |  |  | 0.629 |
| <2 | 59739090(28.78) | 61112716(25.83) | 64014411(26.02) | 69356332(27.57) | 254222549(27) |  |
| 2-4 | 55341548(26.66) | 63732115(26.94) | 70654251(28.72) | 73220243(29.11) | 262948158(27.92) |  |
| ≥4 | 77986378(37.57) | 98053975(41.45) | 95573570(38.85) | 93245969(37.07) | 364859892(38.74) |  |
| Race (%) |  |  |  |  |  | ＜0.001 |
| Mexican American | 21619218(10.41) | 22988778(9.72) | 22143673(9) | 19458081(7.74) | 86209750(9.15) |  |
| Other Hispanic | 12655654(6.10) | 14628908(6.18) | 14852291(6.04) | 15832817(6.29) | 57969670(6.16) |  |
| Non-Hispanic White | 112439475(54.16) | 158027410(66.80) | 170931793(69.49) | 180836295(71.89) | 622234972(66.08) |  |
| Non-Hispanic Black | 41956349(20.21) | 24451674(10.34) | 21195355(8.62) | 19520270(7.76) | 107123648(11.38) |  |
| Other Race | 18931734(9.12) | 16480222(6.97) | 16872752(6.86) | 15881890(6.31) | 68166598(7.24) |  |
| Smoke (%) |  |  |  |  |  | ＜0.001 |
| No | 123620666(59.55) | 141131382(59.66) | 140867387(57.26) | 122908535(48.86) | 528527971(56.12) |  |
| Yes | 83981763(40.45) | 95445609(40.34) | 105128476(42.74) | 128620818(51.14) | 413176667(43.88) |  |
| Drink (%) |  |  |  |  |  | 0.535 |
| No | 156036882(75.16) | 178341775(75.38) | 186845737(75.95) | 190153246(75.60) | 711377640(75.54) |  |
| Yes | 38098458(18.35) | 43754273(18.49) | 48520206(19.72) | 49041972(19.50) | 179414909(19.05) |  |
| SBP if or not treated (%) |  |  |  |  |  | 0.185 |
| No | 179203932(86.32) | 200246433(84.64) | 209342619(85.10) | 208957638(83.07) | 797750622(84.71) |  |
| Yes | 28398497(13.68) | 36330559(15.36) | 36653245(14.90) | 42571715(16.93) | 143954016(15.29) |  |
| T2DM (%) |  |  |  |  |  | 0.364 |
| No | 112358169(54.12) | 130463106(55.15) | 131140664(53.31) | 141428657(56.23) | 515390596(54.73) |  |
| Yes | 95244261(45.88) | 106113885(44.85) | 114855199(46.69) | 110100697(43.77) | 426314042(45.27) |  |
| BMI (kg/m2) (mean±SD) | 27.79±5.49 | 27.92±5.71 | 28.75±6.22 | 28.82±6.78 | 28.35±6.12 | ＜0.001 |
| Underweight | 2115602(1.02) | 4000032(1.69) | 3411186(1.39) | 4415719(1.76) | 13942539(1.48) | ＜0.001 |
| Normal weight | 66740001(32.15) | 74253124(31.39) | 67786122(27.56) | 74716014(29.70) | 283495261(30.10) |  |
| Overweight | 49714563(23.95) | 52891042(22.36) | 54102681(21.99) | 51620270(20.52) | 208328556(22.12) |  |
| Obese | 87437055(42.12) | 104842198(44.32) | 119204145(48.46) | 118405386(47.07) | 429888784(45.65) |  |
| Energy (kcal) (mean±SD) | 2239.35±906.75 | 2240.15±830.64 | 2217.49±859.18 | 2190.60±911.30 | 2220.73±877.11 | 0.497 |
| PA (MET-minutes/week) (mean±SD) | 4190.33±7853.85 | 3278.71±5639.79 | 3807.55±16725.20 | 2832.63±8434.77 | 3501.47±10658.25 | ＜0.001 |
| Vigorous | 50810037(24.47) | 66206314(27.99) | 74630257(30.34) | 85624110(34.04) | 277270718(29.44) | ＜0.001 |
| Moderate | 67201287(32.37) | 80394598(33.98) | 79713021(32.40) | 78639620(31.26) | 305948526(32.49) |  |
| Light | 60828174(29.30) | 54668030(23.11) | 53878631(21.90) | 48187629(19.16) | 217562464(23.10) |  |
| TC (mg/dL) (mean±SD) | 198.40±39.31 | 199.76±40.20 | 200.71±38.51 | 200.85±39.39 | 200.00±39.36 | 0.285 |
| HDL (mg/dL) (mean±SD) | 54.50±16.78 | 53.50±16.07 | 53.10±16.18 | 53.45±15.71 | 53.60±16.17 | 0.157 |
| LDL (mg/dL) (mean±SD) | 118.95±33.89 | 120.08±35.05 | 120.09±33.04 | 120.66±34.61 | 119.98±34.16 | 0.580 |
| SBP (mm Hg) (mean±SD) | 120.04±15.91 | 119.93±15.81 | 120.53±16.42 | 121.42±16.47 | 120.51±16.18 | 0.054 |
| Ln(SII) (mean±SD) | 5.48±0.33 | 5.96±0.09 | 6.29±0.10 | 6.77±0.26 | 6.16±0.51 |  |
| FRS (mean±SD) | 3.93±6.59 | 4.12±6.38 | 4.55±7.49 | 5.04±7.26 | 4.44±6.98 | ＜0.001 |
| Low 10-year CVD risk | 186687561(89.93) | 210673971(89.05) | 218770227(88.93) | 217401364(86.43) | 833533123(88.51) | 0.016 |
| Intermediate and high 10-year CVD risk. | 20914869(10.07) | 25903021(10.95) | 27225637(11.07) | 34127989(13.57) | 108171515(11.49) |  |
| Abbreviations: SD, standard deviation; PIR, poverty income ratio; SBP, systolic blood pressure; T2DM, type 2 diabetes mellitus; BMI, body mass index; PA, physical activity; MET, metabolic equivalent; TC, total cholesterol; HDL, high-density lipoprotein; LDL, low-density lipoprotein; Ln(SII), Ln-transformed SII; SII, systemic immune-inflammation index; FRS, Framingham cardiovascular risk scores; CVD, cardiovascular disease. | | | | | | |
|  |  |  |  |  |  |  |
|  |  |  |  |  |  |  |

| **Supplemental Table2. Associations of Ln(SII) or Ln(SII) quartiles on intermediate and high 10-year CVD risk by weighted generalized linear models.** | | | | | | | |
| --- | --- | --- | --- | --- | --- | --- | --- |
| factor | Crude model | | |  | Adjusted model | | |
|  | OR | 95%CI | *P* value |  | OR | 95%CI | *P* value |
| Ln(SII) | 1.22 | 1.05-1.42 | 0.009 |  | 1.04 | 0.89-1.21 | 0.642 |
|  |  |  |  |  |  |  |  |
| Stratified by Ln(SII) quartiles | 1.11 | 1.04-1.19 | 0.003 |  | 1.03 | 0.96-1.12 | 0.406 |
| Q1 | Ref |  |  |  | Ref |  |  |
| Q2 | 1.10 | 0.85-1.41 | 0.467 |  | 1.07 | 0.79-1.45 | 0.663 |
| Q3 | 1.11 | 0.88-1.39 | 0.367 |  | 1.04 | 0.80-1.35 | 0.789 |
| Q4 | 1.40 | 1.12-1.75 | 0.003 |  | 1.13 | 0.87-1.46 | 0.363 |
| Crude model was not adjusted;  Adjusted model was adjusted for education, marital status, PIR, race, drink, PA, energy, BMI, and LDL.  Abbreviations: Ln(SII), Ln-transformed SII; SII, systemic immune-inflammation index; CVD, cardiovascular disease; OR, odds ratio; Ref, reference; PIR, poverty income ratio; PA, physical activity; BMI, body mass index; LDL, low-density lipoprotein. | | | | | | | |

| **Supplemental Table 3. Associations of Ln(SII) or Ln(SII) quartiles on FRS by weighted generalized linear models in Mexican American.** | | | | | | | |
| --- | --- | --- | --- | --- | --- | --- | --- |
| factor | Crude model | | |  | Adjusted model | | |
|  | OR | 95%CI | *P* value |  | OR | 95%CI | *P* value |
| Ln(SII) | 2.20 | 1.16-4.14 | 0.017 |  | 1.24 | 0.67-2.29 | 0.501 |
|  |  |  |  |  |  |  |  |
| Stratified by Ln(SII) quartiles | 1.45 | 1.11-1.90 | 0.008 |  | 1.15 | 0.88-1.50 | 0.319 |
| Q1 | Ref |  |  |  | Ref |  |  |
| Q2 | 1.90 | 0.87-4.18 | 0.113 |  | 1.75 | 0.81-3.79 | 0.159 |
| Q3 | 1.87 | 0.76-4.60 | 0.178 |  | 1.70 | 0.66-4.40 | 0.278 |
| Q4 | 3.51 | 1.59-7.76 | 0.003 |  | 1.58 | 0.71-3.54 | 0.270 |
| Crude model was not adjusted;  Adjusted model was adjusted for education, marital status, PIR, drink, PA, energy, BMI, and LDL.  Abbreviations: Ln(SII), Ln-transformed SII; SII, systemic immune-inflammation index; FRS, Framingham cardiovascular risk scores; OR, odds ratio; CI, confidence interval; Ref, reference; PIR, poverty income ratio; PA, physical activity; BMI, body mass index; LDL, low-density lipoprotein. | | | | | | | |

| **Supplemental Table 4. Associations of Ln(SII) or Ln(SII) quartiles on intermediate and high 10-year CVD risk by weighted generalized linear models in Mexican American.** | | | | | | | |
| --- | --- | --- | --- | --- | --- | --- | --- |
| factor | Crude model | | |  | Adjusted model | | |
|  | OR | 95%CI | *P* value |  | OR | 95%CI | *P* value |
| Ln(SII) | 1.18 | 0.81-1.72 | 0.396 |  | 0.84 | 0.58-1.22 | 0.365 |
|  |  |  |  |  |  |  |  |
| Stratified by Ln(SII) quartiles | 1.12 | 0.95-1.33 | 0.179 |  | 0.97 | 0.82-1.15 | 0.733 |
| Q1 | Ref |  |  |  | Ref |  |  |
| Q2 | 1.31 | 0.77-2.22 | 0.325 |  | 1.22 | 0.68-2.17 | 0.512 |
| Q3 | 1.21 | 0.66-2.21 | 0.543 |  | 1.09 | 0.56-2.11 | 0.800 |
| Q4 | 1.53 | 0.91-2.57 | 0.115 |  | 0.95 | 0.56-1.62 | 0.857 |
| Crude model was not adjusted;  Adjusted model was adjusted for education, marital status, PIR, drink, PA, energy, BMI, and LDL.  Abbreviations: Ln(SII), Ln-transformed SII; SII, systemic immune-inflammation index; CVD, cardiovascular disease; OR, odds ratio; CI, confidence interval; Ref, reference; PIR, poverty income ratio; PA, physical activity; BMI, body mass index; LDL, low-density lipoprotein. | | | | | | | |

| **Supplemental Table 5. Associations of Ln(SII) or Ln(SII) quartiles on FRS by weighted generalized linear models in Other Hispanic.** | | | | | | | |
| --- | --- | --- | --- | --- | --- | --- | --- |
| factor | Crude model | | |  | Adjusted model | | |
|  | OR | 95%CI | *P* value |  | OR | 95%CI | *P* value |
| Ln(SII) | 0.58 | 0.18-1.85 | 0.357 |  | 0.94 | 0.37-2.37 | 0.895 |
|  |  |  |  |  |  |  |  |
| Stratified by Ln(SII) quartiles | 0.75 | 0.45-1.24 | 0.263 |  | 0.94 | 0.63-1.40 | 0.759 |
| Q1 | Ref |  |  |  | Ref |  |  |
| Q2 | 0.24 | 0.05-1.12 | 0.073 |  | 0.34 | 0.11-1.03 | 0.061 |
| Q3 | 0.50 | 0.08-3.20 | 0.467 |  | 0.61 | 0.15-2.48 | 0.491 |
| Q4 | 0.28 | 0.05-1.52 | 0.143 |  | 0.63 | 0.17-2.27 | 0.478 |
| Crude model was not adjusted;  Adjusted model was adjusted for education, marital status, PIR, drink, PA, energy, BMI, and LDL.  Abbreviations: Ln(SII), Ln-transformed SII; SII, systemic immune-inflammation index; FRS, Framingham cardiovascular risk scores; OR, odds ratio; CI, confidence interval; Ref, reference; PIR, poverty income ratio; PA, physical activity; BMI, body mass index; LDL, low-density lipoprotein. | | | | | | | |

| **Supplemental Table 6. Associations of Ln(SII) or Ln(SII) quartiles on intermediate and high 10-year CVD risk by weighted generalized linear models in Other Hispanic.** | | | | | | | |
| --- | --- | --- | --- | --- | --- | --- | --- |
| factor | Crude model | | |  | Adjusted model | | |
|  | OR | 95%CI | *P* value |  | OR | 95%CI | *P* value |
| Ln(SII) | 0.94 | 0.46-1.92 | 0.860 |  | 1.39 | 0.73-2.65 | 0.327 |
|  |  |  |  |  |  |  |  |
| Stratified by Ln(SII) quartiles | 0.94 | 0.72-1.23 | 0.664 |  | 1.10 | 0.82-1.48 | 0.526 |
| Q1 | Ref |  |  |  | Ref |  |  |
| Q2 | 0.71 | 0.35-1.44 | 0.346 |  | 0.83 | 0.41-1.71 | 0.623 |
| Q3 | 0.72 | 0.35-1.48 | 0.373 |  | 0.85 | 0.44-1.62 | 0.618 |
| Q4 | 0.81 | 0.37-1.77 | 0.599 |  | 1.31 | 0.58-2.96 | 0.526 |
| Crude model was not adjusted;  Adjusted model was adjusted for education, marital status, PIR, drink, PA, energy, BMI, and LDL.  Abbreviations: Ln(SII), Ln-transformed SII; SII, systemic immune-inflammation index; CVD, cardiovascular disease; OR, odds ratio; CI, confidence interval; Ref, reference; PIR, poverty income ratio; PA, physical activity; BMI, body mass index; LDL, low-density lipoprotein. | | | | | | | |

| **Supplemental Table 7. Associations of Ln(SII) or Ln(SII) quartiles on FRS by weighted generalized linear models in Non-Hispanic White.** | | | | | | | |
| --- | --- | --- | --- | --- | --- | --- | --- |
| factor | Crude model | | |  | Adjusted model | | |
|  | OR | 95%CI | *P* value |  | OR | 95%CI | *P* value |
| Ln(SII) | 3.07 | 1.91-4.92 | <0.001 |  | 1.72 | 1.13-2.60 | 0.012 |
|  |  |  |  |  |  |  |  |
| Stratified by Ln(SII) quartiles | 1.72 | 1.39-2.13 | <0.001 |  | 1.30 | 1.08-1.58 | 0.007 |
| Q1 | Ref |  |  |  | Ref |  |  |
| Q2 | 1.66 | 0.85-3.24 | 0.143 |  | 1.41 | 0.73-2.72 | 0.312 |
| Q3 | 2.73 | 1.41-5.31 | 0.004 |  | 1.67 | 0.95-2.94 | 0.077 |
| Q4 | 5.10 | 2.63-9.87 | <0.001 |  | 2.30 | 1.27-4.16 | 0.007 |
| Crude model was not adjusted;  Adjusted model was adjusted for education, marital status, PIR, drink, PA, energy, BMI, and LDL.  Abbreviations: Ln(SII), Ln-transformed SII; SII, systemic immune-inflammation index; FRS, Framingham cardiovascular risk scores; OR, odds ratio; CI, confidence interval; Ref, reference; PIR, poverty income ratio; PA, physical activity; BMI, body mass index; LDL, low-density lipoprotein. | | | | | | | |

| **Supplemental Table 8. Associations of Ln(SII) or Ln(SII) quartiles on intermediate and high 10-year CVD risk by weighted generalized linear models in Non-Hispanic White.** | | | | | | | |
| --- | --- | --- | --- | --- | --- | --- | --- |
| factor | Crude model | | |  | Adjusted model | | |
|  | OR | 95%CI | *P* value |  | OR | 95%CI | *P* value |
| Ln(SII) | 1.40 | 1.13-1.74 | 0.003 |  | 1.05 | 0.94-1.17 | 0.398 |
|  |  |  |  |  |  |  |  |
| Stratified by Ln(SII) quartiles | 1.18 | 1.06-1.31 | 0.002 |  | 1.06 | 0.98-1.14 | 0.162 |
| Q1 | Ref |  |  |  | Ref |  |  |
| Q2 | 1.23 | 0.83-1.82 | 0.302 |  | 1.11 | 0.70-1.76 | 0.669 |
| Q3 | 1.30 | 0.92-1.85 | 0.145 |  | 1.12 | 0.75-1.66 | 0.584 |
| Q4 | 1.68 | 1.19-2.38 | 0.004 |  | 1.18 | 0.80-1.74 | 0.406 |
| Crude model was not adjusted;  Adjusted model was adjusted for education, marital status, PIR, drink, PA, energy, BMI, and LDL.  Abbreviations: Ln(SII), Ln-transformed SII; SII, systemic immune-inflammation index; CVD, cardiovascular disease; OR, odds ratio; CI, confidence interval; Ref, reference; PIR, poverty income ratio; PA, physical activity; BMI, body mass index; LDL, low-density lipoprotein. | | | | | | | |

| **Supplemental Table 9. Associations of Ln(SII) or Ln(SII) quartiles on FRS by weighted generalized linear models in Non-Hispanic Black.** | | | | | | | |
| --- | --- | --- | --- | --- | --- | --- | --- |
| factor | Crude model | | |  | Adjusted model | | |
|  | OR | 95%CI | *P* value |  | OR | 95%CI | *P* value |
| Ln(SII) | 1.85 | 1.02-3.33 | 0.045 |  | 1.73 | 0.99-3.04 | 0.058 |
|  |  |  |  |  |  |  |  |
| Stratified by Ln(SII) quartiles | 1.31 | 0.98-1.75 | 0.076 |  | 1.32 | 0.99-1.77 | 0.065 |
| Q1 | Ref |  |  |  | Ref |  |  |
| Q2 | 1.00 | 0.47-2.12 | 0.996 |  | 0.98 | 0.49-1.95 | 0.952 |
| Q3 | 2.12 | 0.82-5.44 | 0.123 |  | 2.34 | 0.91-6.05 | 0.082 |
| Q4 | 1.95 | 0.79-4.83 | 0.153 |  | 1.91 | 0.75-4.89 | 0.178 |
| Crude model was not adjusted;  Adjusted model was adjusted for education, marital status, PIR, drink, PA, energy, BMI, and LDL.  Abbreviations: Ln(SII), Ln-transformed SII; SII, systemic immune-inflammation index; FRS, Framingham cardiovascular risk scores; OR, odds ratio; CI, confidence interval; Ref, reference; PIR, poverty income ratio; PA, physical activity; BMI, body mass index; LDL, low-density lipoprotein. | | | | | | | |

| **Supplemental Table 10. Associations of Ln(SII) or Ln(SII) quartiles on intermediate and high 10-year CVD risk by weighted generalized linear models in Non-Hispanic Black.** | | | | | | | |
| --- | --- | --- | --- | --- | --- | --- | --- |
| factor | Crude model | | |  | Adjusted model | | |
|  | OR | 95%CI | *P* value |  | OR | 95%CI | *P* value |
| Ln(SII) | 1.12 | 0.89-1.41 | 0.346 |  | 1.11 | 0.86-1.44 | 0.438 |
|  |  |  |  |  |  |  |  |
| Stratified by Ln(SII) quartiles | 1.03 | 0.91-1.16 | 0.636 |  | 1.04 | 0.91-1.19 | 0.563 |
| Q1 | Ref |  |  |  | Ref |  |  |
| Q2 | 1.03 | 0.73-1.45 | 0.862 |  | 1.00 | 0.66-1.51 | 0.993 |
| Q3 | 1.11 | 0.79-1.54 | 0.555 |  | 1.19 | 0.81-1.77 | 0.381 |
| Q4 | 1.07 | 0.73-1.57 | 0.739 |  | 1.07 | 0.69-1.65 | 0.772 |
| Crude model was not adjusted;  Adjusted model was adjusted for education, marital status, PIR, drink, PA, energy, BMI, and LDL.  Abbreviations: Ln(SII), Ln-transformed SII; SII, systemic immune-inflammation index; CVD, cardiovascular disease; OR, odds ratio; CI, confidence interval; Ref, reference; PIR, poverty income ratio; PA, physical activity; BMI, body mass index; LDL, low-density lipoprotein. | | | | | | | |

| **Supplemental Table 11. Associations of Ln(SII) or Ln(SII) quartiles on FRS by weighted generalized linear models in Other Race.** | | | | | | | |
| --- | --- | --- | --- | --- | --- | --- | --- |
| factor | Crude model | | |  | Adjusted model | | |
|  | OR | 95%CI | *P* value |  | OR | 95%CI | *P* value |
| Ln(SII) | 0.60 | 0.25-1.42 | 0.246 |  | 0.55 | 0.24-1.28 | 0.172 |
|  |  |  |  |  |  |  |  |
| Stratified by Ln(SII) quartiles | 0.89 | 0.59-1.32 | 0.553 |  | 0.86 | 0.58-1.29 | 0.479 |
| Q1 | Ref |  |  |  | Ref |  |  |
| Q2 | 0.71 | 0.21-2.41 | 0.590 |  | 0.48 | 0.13-1.76 | 0.276 |
| Q3 | 0.45 | 0.13-1.54 | 0.208 |  | 0.48 | 0.14-1.62 | 0.243 |
| Q4 | 0.80 | 0.23-2.80 | 0.733 |  | 0.64 | 0.18-2.30 | 0.499 |
| Crude model was not adjusted;  Adjusted model was adjusted for education, marital status, PIR, drink, PA, energy, BMI, and LDL.  Abbreviations: Ln(SII), Ln-transformed SII; SII, systemic immune-inflammation index; FRS, Framingham cardiovascular risk scores; OR, odds ratio; CI, confidence interval; Ref, reference; PIR, poverty income ratio; PA, physical activity; BMI, body mass index; LDL, low-density lipoprotein. | | | | | | | |

| **Supplemental Table 12. Associations of Ln(SII) or Ln(SII) quartiles on intermediate and high 10-year CVD risk by weighted generalized linear models in Other Race.** | | | | | | | |
| --- | --- | --- | --- | --- | --- | --- | --- |
| factor | Crude model | | |  | Adjusted model | | |
|  | OR | 95%CI | *P* value |  | OR | 95%CI | *P* value |
| Ln(SII) | 0.60 | 0.36-0.99 | 0.050 |  | 0.46 | 0.27-0.79 | 0.007 |
|  |  |  |  |  |  |  |  |
| Stratified by Ln(SII) quartiles | 0.91 | 0.71-1.16 | 0.432 |  | 0.81 | 0.62-1.06 | 0.132 |
| Q1 | Ref |  |  |  | Ref |  |  |
| Q2 | 0.96 | 0.55-1.67 | 0.893 |  | 0.94 | 0.45-1.94 | 0.863 |
| Q3 | 0.60 | 0.30-1.17 | 0.139 |  | 0.41 | 0.19-0.90 | 0.031 |
| Q4 | 0.85 | 0.41-1.77 | 0.669 |  | 0.66 | 0.29-1.48 | 0.319 |
| Crude model was not adjusted;  Adjusted model was adjusted for education, marital status, PIR, drink, PA, energy, BMI, and LDL.  Abbreviations: Ln(SII), Ln-transformed SII; SII, systemic immune-inflammation index; CVD, cardiovascular disease; OR, odds ratio; CI, confidence interval; Ref, reference; PIR, poverty income ratio; PA, physical activity; BMI, body mass index; LDL, low-density lipoprotein. | | | | | | | |
